# Mm-VitnNet: a gated image-text interaction network for soybean salt tolerance recognition using chlorophyll fluorescence phenotypes

**DOI:** 10.3389/fpls.2026.1721287

**Published:** 2026-03-17

**Authors:** Wenxiang Liang, Xiaoyan Zhang, Ziqiu Luo, Qingyang Li, Hao Wang, Yixin Feng, Licheng Zhao, Ziyan Lu, Xiaotian Yuan, Xiouxiou Zhou, Lu Huang, Xin Chen, Zhe Yan, Shangbing Gao, Chenchen Xue

**Affiliations:** 1 Trusted Firmware and Intelligent Software Laboratory, Huaiyin Institute of Technology, Huai’an, China; 2 Institute of Economic Crops, Jiangsu Academy of Agricultural Sciences, Nanjing, China; 3 College of Life Sciences, Jiangsu University, Zhenjiang, China; 4 Institute of Crop Sciences, Chinese Academy of Agricultural Sciences, Beijing, China

**Keywords:** chlorophyll fluorescence imaging, convolutional neural networks, gated mechanism, salt tolerance level, soybean salt tolerance identification method, transformer

## Abstract

Traditional methods for identifying salt tolerance levels in soybean varieties are often cumbersome, time-consuming, and labor-intensive. These challenges are further exacerbated by the limited utility of chlorophyll fluorescence imaging phenotype data, which are insufficiently diverse and difficult to analyze. Additionally, the corresponding parameter text data have not been fully explored and utilized. In this study, salt stress experiments were conducted on 178 soybean varieties, and a multimodal dataset comprising chlorophyll fluorescence images and corresponding textual data was constructed using a chlorophyll fluorescence imaging instrument. A novel gated mechanism network for learnable image-text interaction (Mm-VitnNet) is proposed, which enables global cross-modal interaction between image and text data. The model introduces a gated mechanism to dynamically regulate the fusion intensity of cross-modal information and incorporates two learnable tokens that focus on feature learning for each individual modality. This approach effectively mitigates interference between modalities while preserving modality-specific features, thereby enhancing model performance. The proposed model demonstrates an accuracy rate of 98.97%, significantly outperforming typical models: it improves by 1.09 and 2.33 percentage points compared to CNN-based models such as EfficientNetV2-s (97.88%) and MobileNetV2 (96.64%), respectively, and by 3.21 and 2.60 percentage points compared to Transformer-based Swin Transformer_tiny (95.76%) and hybrid models like MobileViT_S (96.37%), respectively. The model has 10.22M parameters and a computational cost (FLOPs) of 1.84G, which is significantly lower than models like VGG and ResNet50, and only slightly higher than some lightweight CNNs, achieving an effective balance between accuracy and efficiency. The improved model demonstrates notable performance in identifying samples with varying salt tolerance levels, even under limited computational resources, ensuring reliable classification performance. Moreover, this multimodal non-destructive identification method based on chlorophyll fluorescence technology offers an efficient and feasible approach for assessing the salt tolerance levels of soybeans, while also advancing agricultural phenotyping towards greater precision and intelligence.

## 1 Introduction

Soybean (*Glycine max* (L.) Merr.) is a herbaceous annual plant belonging to the Fabaceae family, recognized for its significant value in various fields. In the food sector, it provides high-quality plant protein essential for the human diet and serves as a vital raw material for producing soybean-derived products such as tofu, soy milk, and fermented soy foods. In the industrial field, its oil components are utilized in the production of biodiesel and various industrial products. In agriculture, soybean, through symbiosis with rhizobia, achieves biological nitrogen fixation, effectively enhancing soil fertility and serving as a critical crop in crop rotation systems. Additionally, in animal husbandry, it is a key component of high-quality feed, supplying abundant protein for livestock and poultry farming (Arshad et al., 2025; Deng et al., 2025). However, soil salinization is a global resource and ecological challenge. Despite the widespread cultivation of soybeans and high demand, arable land is limited. In China, saline-alkali soils span roughly 99.13 million hectares—ranking third in the world—and represent about 10% of the nation’s total land area. These soils are mainly distributed in the northeastern, northern, and northwestern regions of China (Zhu et al., 2018; Liu et al., 2023; Yao et al., 2025). To meet the growing demand for soybeans, numerous breeding experiments have been conducted, resulting in significant progress in high-yield research, variety improvement, and resistance enhancement (Chen et al., 2025; Miranda et al., 2025; Sun et al., 2025). Despite this, salt-tolerant soybean varieties remain scarce.

The assessment of salt tolerance in soybean germplasm resources is crucial for breeding varieties that can withstand saline conditions (Han et al., 2023). Traditional approaches for assessing salt tolerance in soybean germplasm face considerable limitations regarding efficiency, accuracy, and the capacity for genetic analysis. In practice, phenotypic identification and physiological-biochemical index measurements under salt stress can directly reveal the salt tolerance performance of soybean in real-world conditions (Han et al., 2025). Nevertheless, traditional methods are highly susceptible to environmental factors. Variations in climate conditions and soil salinity across years and regions can lead to inconsistent salt tolerance performance in the same variety, reducing the repeatability and reliability of identification results. Traditional approaches to assess salt tolerance in soybean germplasm primarily consist of two categories: manual evaluation by experienced experts and machine-based image analysis (Sun et al., 2017; Chen et al., 2024). While manual evaluation relies on expert knowledge and observation of various phenotypic parameters, it is inefficient, labor-intensive, and prone to subjectivity, especially when assessing large-scale plant populations. This creates a “phenotyping bottleneck,” characterized by low throughput, high costs, and labor-intensive processing in traditional crop phenotyping (Wang et al., 2022).

With the rapid development of deep learning technologies, their applications in agriculture have been continuously expanding, promoting the transformation of agricultural production from traditional experience-based practices toward precision and intelligence. Chlorophyll fluorescence technology enables non-destructive monitoring of the physiological status of plant photosynthesis by detecting fluorescence signals emitted by chlorophyll upon light excitation. To ensure the accuracy and comparability of fluorescence parameter measurements, dark adaptation is typically performed prior to data acquisition, allowing photosystem II (PSII) reaction centers to return to their initial open state and thereby ensuring the reliability of parameters such as the maximum quantum yield of photochemistry (QY_max). This technique has been widely applied in plant physiological studies, crop growth monitoring, and ecological vegetation health assessment. Cheng et al. (Cheng et al., 2025) integrated chlorophyll fluorescence imaging (CFI) with hyperspectral imaging (HSI) to assess the natural aging process of pears during the transition from green to yellow under different storage conditions. Lee et al. (Lee et al., 2025) employed a pulse-amplitude-modulated fluorometer to screen chlorophyll fluorescence indices, enabling rapid diagnosis before the appearance of disease symptoms. Yang et al. (Yang Y. et al., 2025) combined hyperspectral and chlorophyll fluorescence imaging data to construct a support vector machine model for identifying wheat cultivars with different levels of drought resistance, achieving an accuracy of 97.33%. Yao et al. (Yao et al., 2021) proposed a data-driven signal dynamics prediction model (SLSTM-TCNN) to characterize the temporal dependence between electrical signals and NaCl stress. Qi et al. (Qi et al., 2024) collected plant electrical signals under different soil moisture gradients and designed a lightweight convolutional neural network, PlantNet, which effectively classified stress responses. These studies mainly establish stress response models based on a single sensing modality (e.g., spectral data or electrical signals), focusing on the quantitative analysis of one-dimensional indicators. To overcome the limitations of single-modality information, some studies have begun to explore multi-source information fusion approaches. Yang et al. (Yang M. et al., 2025) integrated spectral information with chlorophyll fluorescence physiological parameters to develop a spectral-feature-based heterogeneous inversion model for early crop disease warning. Chen et al. (Chen et al., 2024) selected the chlorophyll fluorescence parameter Ft_D3 and combined it with an improved ResNet50 transfer learning model to achieve early detection of cucumber downy mildew, with an accuracy of 94.76%. Jiang et al. (Jiang et al., 2025) proposed a multi-task convolutional neural network (MT-CNN) based on ultraviolet fluorescence spectroscopy to simultaneously predict multiple quality indicators of rapeseed oil. Chu et al. (Zhang et al., 2022) fused hyperspectral imaging and chlorophyll fluorescence imaging data to propose an end-to-end deep fusion model, achieving a detection accuracy of 97.7%. However, research on salt stress tolerance identification still primarily focuses on image-level features, and chlorophyll fluorescence parameter information has not yet been fully exploited.

The multimodal non-destructive identification method based on chlorophyll fluorescence technology significantly shortens the assessment cycle for salt tolerance levels in soybeans while enhancing the accuracy and efficiency of the identification process. It achieves a good balance between recognition accuracy and model complexity under limited computational resources. This method provides a feasible technical path for the rapid screening of salt tolerance in large-scale germplasm resources and facilitates intelligent phenotypic analysis. It supports the efficient selection of salt-tolerant soybean germplasm, improves the survival rate of crops in saline-alkali soils, and ensures yield stability. Furthermore, it holds practical reference value for evaluating crop salt tolerance and precision breeding. The main contributions of this study are as follows:

1. This research utilizes two types of multimodal data: chlorophyll fluorescence text and images. Given that the text data corresponds to the textual information of chlorophyll fluorescence images, it is more complex and contains more redundant information. Principal Component Analysis (PCA) is applied to reduce the dimensionality of the text data, and a cross-attention mechanism is employed to facilitate interaction between the image and text, using image features to focus on text features while constructing an image gating mechanism to determine the extent of text feature information to be fused.
2. Due to the relatively uniform distribution of attention, effectively focusing on truly long-range dependent feature information can be challenging. This study introduces two learnable tokens to capture the long-distance dependencies of both image and text data, helping to learn the long-range feature information from both modalities.
3. The data in this study are collected using a FluorCam multispectral fluorescence imaging system, and a novel GCF-tp multimodal dataset for salt tolerance identification in soybeans is established. This dataset includes textual data corresponding to all parameters of soybean seedling leaves and images related to maximum quantum efficiency of photochemistry (QY_max). The combination of image and text data enables a more comprehensive assessment of salt tolerance in soybean seedling leaves.

## 2 Materials and methods

### 2.1 Related technical

**Figure 1:**
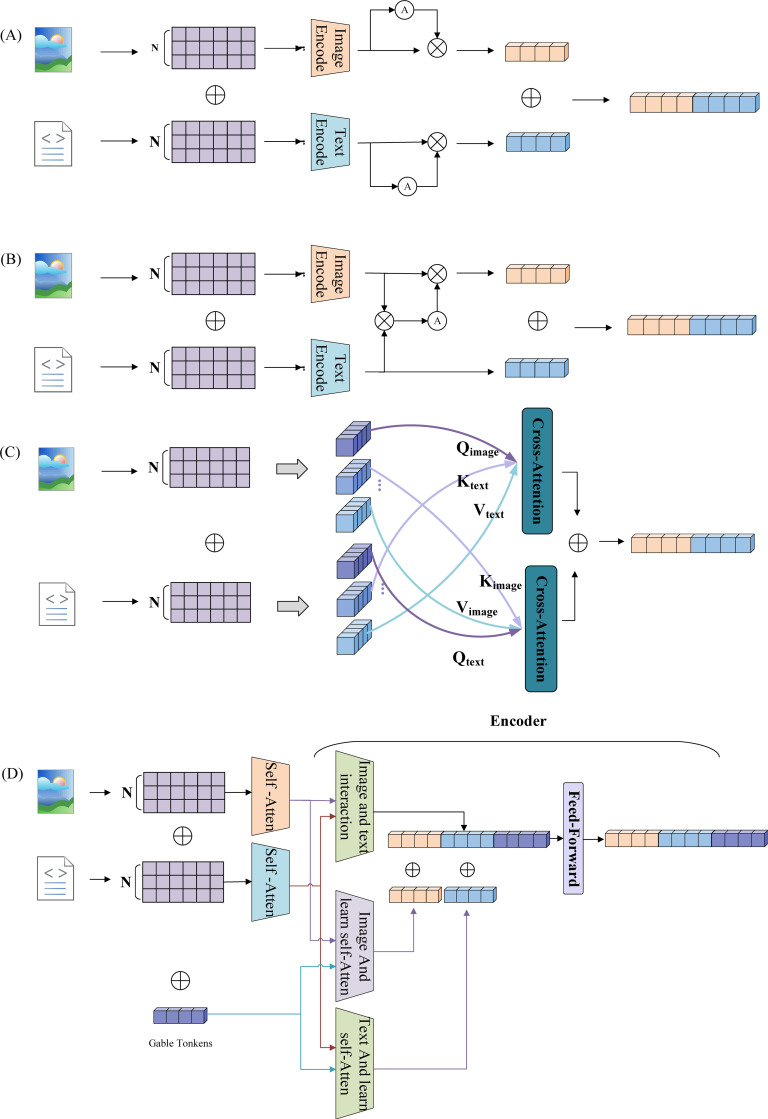
Illustrates various multimodal fusion methods utilizing different attention mechanisms. **(A)** Depicts the attention mechanism that emphasizes intra-modal relationships. **(B)** Highlights the attention mechanism focusing on inter-modal relationships. **(C)** Presents a Transformer-based architecture incorporating both intra-modal self-attention and inter-modal cross-attention. Finally, **(D)** Showcases a multimodal fusion method based on learnable long-distance cross-attention.

A network is constructed using the multi-head self-attention mechanism within the Transformer encoder (Vaswani et al., 2017) to address multimodal fusion in soybean salt tolerance recognition. Unlike the local convolution operations of CNNs, the self-attention mechanism captures dependencies between elements at any position in the sequence, leveraging global information to handle complex semantic relationships and enhance feature expressiveness. Different modalities exhibit distinct characteristics: images consist of continuous pixel matrices with greater detail, while text represents discrete symbolic descriptions of overall information. The primary challenge in multimodal fusion lies in aligning these two modalities. Although their semantic content is related, the differences in underlying features complicate direct matching. Thus, it is necessary to achieve alignment by mapping to a common semantic space. The multimodal fusion methods based on the self-attention mechanism are illustrated in **Figure 1A** focuses on the attention mechanism related to intra-modality relationships, while **Figure 1B** shows the attention mechanism concentrating on inter-modality relationships. **Figure 1C** displays the Transformer-based architecture, including intra-modality self-attention and inter-modality cross-attention. In **Figure 1A**, the model focuses solely on the relationships within each modality before concatenation; **Figure 1B** emphasizes the extraction of features from each modality, identifying and integrating associated features to increase the weight of modality correlations; **Figure 1C** utilizes different modalities as queries, keys, and values in a cross-attention mechanism, facilitating attention calculations among features from different modalities and allowing the model to focus on mutually relevant information (Zhao et al., 2024). This study presents a learnable long-distance cross-attention multimodal fusion approach, illustrated in **Figure 1D**. Initially, self-attention is applied separately to images and text to capture key features within each modality. Then, a cross-modality attention and interaction module enables images and text to “understand” each other’s semantics, associating the “chlorophyll fluorescence of leaves” in images with the “chlorophyll fluorescence parameters of leaves” in text. Learnable tokens are introduced to assist in the aggregation of cross-modal information and global representation learning. By establishing multi-level image-text cross-attention interactions, the model can delve deeper into the semantic associations between images and text. The introduction of learnable global tokens (Gable Tokens) (Huang et al., 2022) further enhances the model’s ability to capture key information and global features in multimodal data, thereby improving performance in multimodal tasks.

### 2.2 Data collection

A total of 178 soybean germplasm resources, originating from different regions and not pre-screened for salt tolerance, were selected as experimental materials. These materials are preserved by the Jiangsu Academy of Agricultural Sciences, with the aim of constructing a representative population exhibiting extensive natural variation. Related experiments are scheduled to take place from 2024 to 2025 at the Jiangsu Academy of Agricultural Sciences. Initially, vermiculite was used for the germination of soybean seeds, followed by cultivation in a 30-well hydroponic box (38.5 × 26.5 × 14.5 cm). Data will be collected using a FlourCam multispectral fluorescence imaging system (manufactured by PSI, Czech Republic).

To determine suitable salt stress conditions, a preliminary gradient experiment was conducted. The concentration gradient was set at 50,100,150,200,and 250 mmol·L^−1^ NaCl, with treatment durations of 3, 5, 7, and 10 days, using the chlorophyll fluorescence phenotypic data of the tested materials as a basis for evaluation. Results indicated that a treatment of 150 mmol·L^−1^ NaCl for 5 days consistently exhibited typical salt stress response characteristics in the tested materials. This condition neither caused insufficient stress effects due to being too low or too short nor resulted in mortality from being too high or too long. Therefore, this configuration was deemed the optimal condition, balancing stress effectiveness with material viability, effectively distinguishing the salt tolerance differences among the various materials.

**Figure 2:**
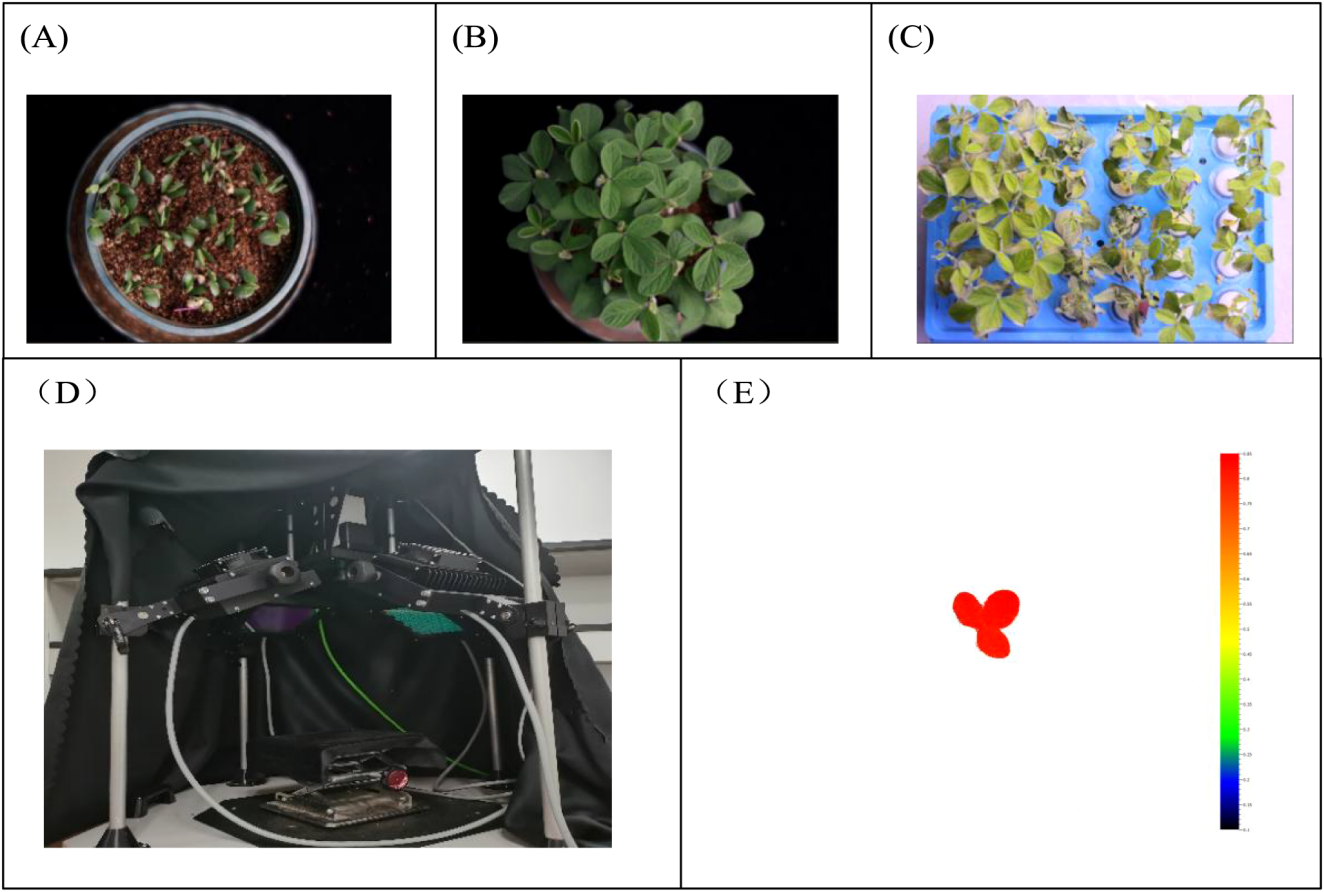
Chlorophyll fluorescence data acquisition process. **(A)** Soil-cultured soybean sprout stage plants. **(B)** Soil-cultured soybean seedling plants. **(C)** Hydroponic soybean seedling-stressed plants. **(D)** FluorCam Open Chlorophyll Fluorescence Imaging System. **(E)** Chlorophyll fluorescence analysis image data.

The detailed cultivation procedures are outlined below:

1. Fill pots with vermiculite and sow seeds according to their respective numbers. After 5 days of growth, as shown in **Figure 2A**, the soybean seedlings develop compound leaves after 12 days, as illustrated in **Figure 2B**, preparing them for subsequent growth in stress conditions.
2. Based on the results of the preliminary experiment, there were small differences in chlorophyll fluorescence values among individual plants in the control group. Therefore, the treatment group was conducted with 10 repetitions, while the control group underwent 3 repetitions. After washing the roots, soybean plants with similar growth status were selected for each variety. The plants were cultivated in distilled water at 24 °C for one day, followed by one day in Hoagland nutrient solution (Coolaber) to acclimatize them to the hydroponic growth environment.
3. In the experimental group, Hoagland nutrient solution and 150 mmol·L^−1^ sodium chloride were added to simulate salt stress, and the plants were grown under salt stress conditions for 5 days, as depicted in **Figure 2C**.
4. The soybean seedlings underwent dark treatment to restore the state of the photosynthetic system and reduce environmental light interference. On the morning of the sixth day, chlorophyll fluorescence status under salt stress was collected using a chlorophyll fluorescence instrument, as shown in **Figure 2D**.
5. The accompanying FluorCam imaging analysis software was initiated to analyze the collected data, as illustrated in **Figure 2E**, saving QY_max chlorophyll fluorescence image data along with all corresponding parameters.

### 2.3 Multimodal data processing

#### 2.3.1 Anomaly detection in textual data

**Figure 3:**
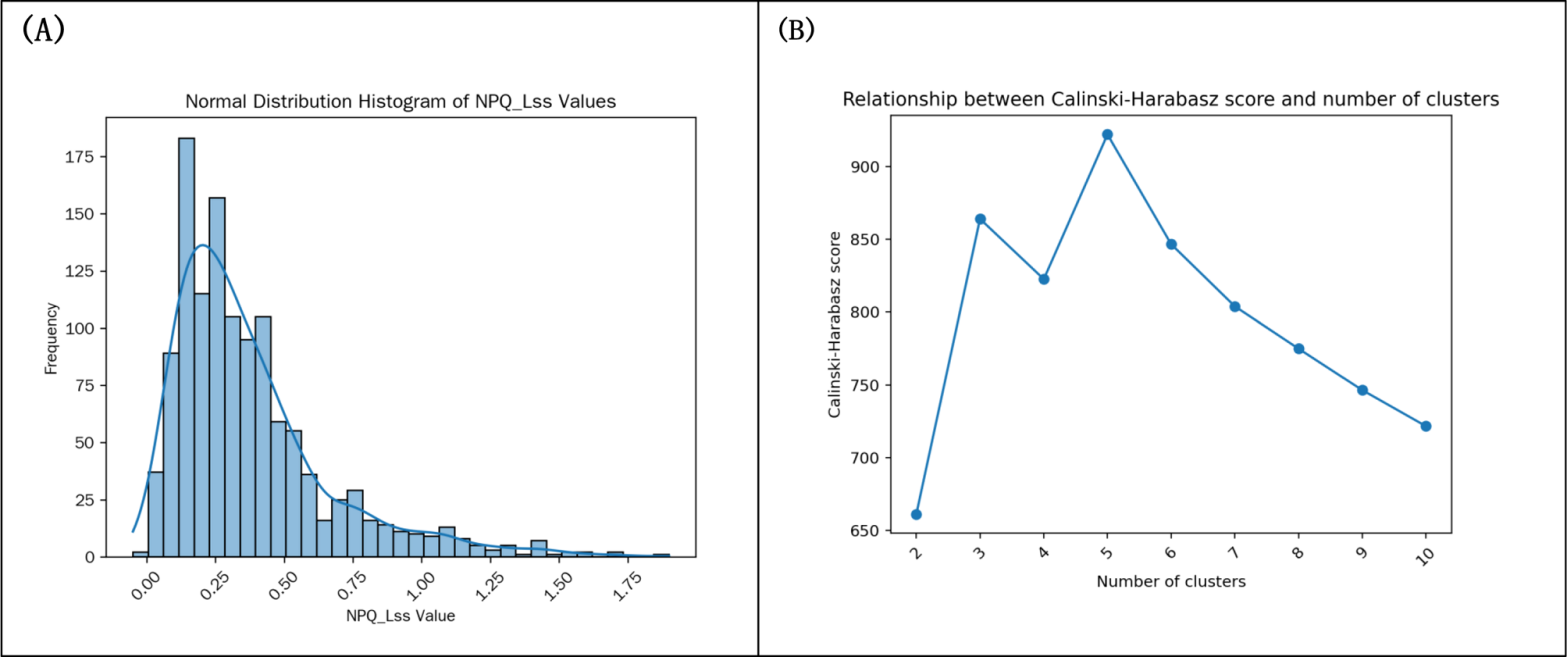
Chlorophyll fluorescence data acquisition process. **(A)** NPQ_Lss normal distribution Plot. **(B)** Line graph of the number of optimal clusters.

Chlorophyll fluorescence parameters effectively characterize the photosynthetic performance of soybeans under salt stress conditions. To construct a multimodal fusion framework, a total of 80 chlorophyll fluorescence parameters were collected. Considering the matching requirements between image and text data, random variations between 0.001 and 0.009 were applied to the text data parameters to augment the dataset. Subsequently, Principal Component Analysis (PCA) (Allahem et al., 2025) was employed for dimensionality reduction, organizing the data into a numerical sequence to serve as “text-like” input. Key parameters include the maximum fluorescence (Fm), which reflects the potential efficiency of light energy conversion at the photosystem II (PSII) reaction center; QY_max, used to assess the capacity for capturing light energy and its conversion to chemical energy; NPQ_Lss, which characterizes the level of non-photochemical quenching and photoprotection mechanisms under stress; qP_Lss, closely related to the rate of electron transfer, with reductions typically indicating suppressed PSII activity under salt stress; and Rfd_Lss, reflecting the open state of the reaction center and light energy utilization efficiency. According to the statistics presented in **Table 1**, the means and medians of QY_max, NPQ_Lss, qP_Lss, and Rfd_Lss are closely aligned, with relatively symmetrical distributions. In contrast, Fm shows a significant difference between its mean and median, with a high standard deviation of 6568.85 and a range of 1612.72 to 31857.48, indicating substantial variability in maximum fluorescence among different samples. The maximum value of NPQ_Lss, at 1.90, deviates significantly from both the mean and quartiles, as illustrated in **Figure 3A**, where the normal distribution plot indicates that only a small number of NPQ_Lss values diverge. To preserve data diversity, these outliers were not removed. Overall, despite the presence of local skewness and a few outliers, the overall distribution accurately reflects the true state of the soybean photosynthetic system under salt stress, providing a reliable basis for further research into the mechanisms underlying soybean salt tolerance and photosynthetic responses.

**Table 1:** Analysis of soybean chlorophyll fluorescence salt tolerance text data.

Measurements	Fm	QY_max	NPQ_Lss	qP_Lss	Rfd_Lss
mean	11797.78	0.49	0.38	0.48	0.79
std	6568.85	0.23	0.29	0.22	0.53
min	1612.72	0.20	-0.05	-0.18	0.12
25%	6302.00	0.29	0.17	0.26	0.36
50%	11347.50	0.43	0.30	0.51	0.57
75%	15768.39	0.71	0.47	0.67	1.18
max	31857.48	0.90	1.90	1.11	2.81
variance	4.31×10^7^	5.24×10^-2^	8.23×10^-2^	4.96×10^-2^	2.85×10^-1^

#### 2.3.2 Image data processing

**Figure 4:**
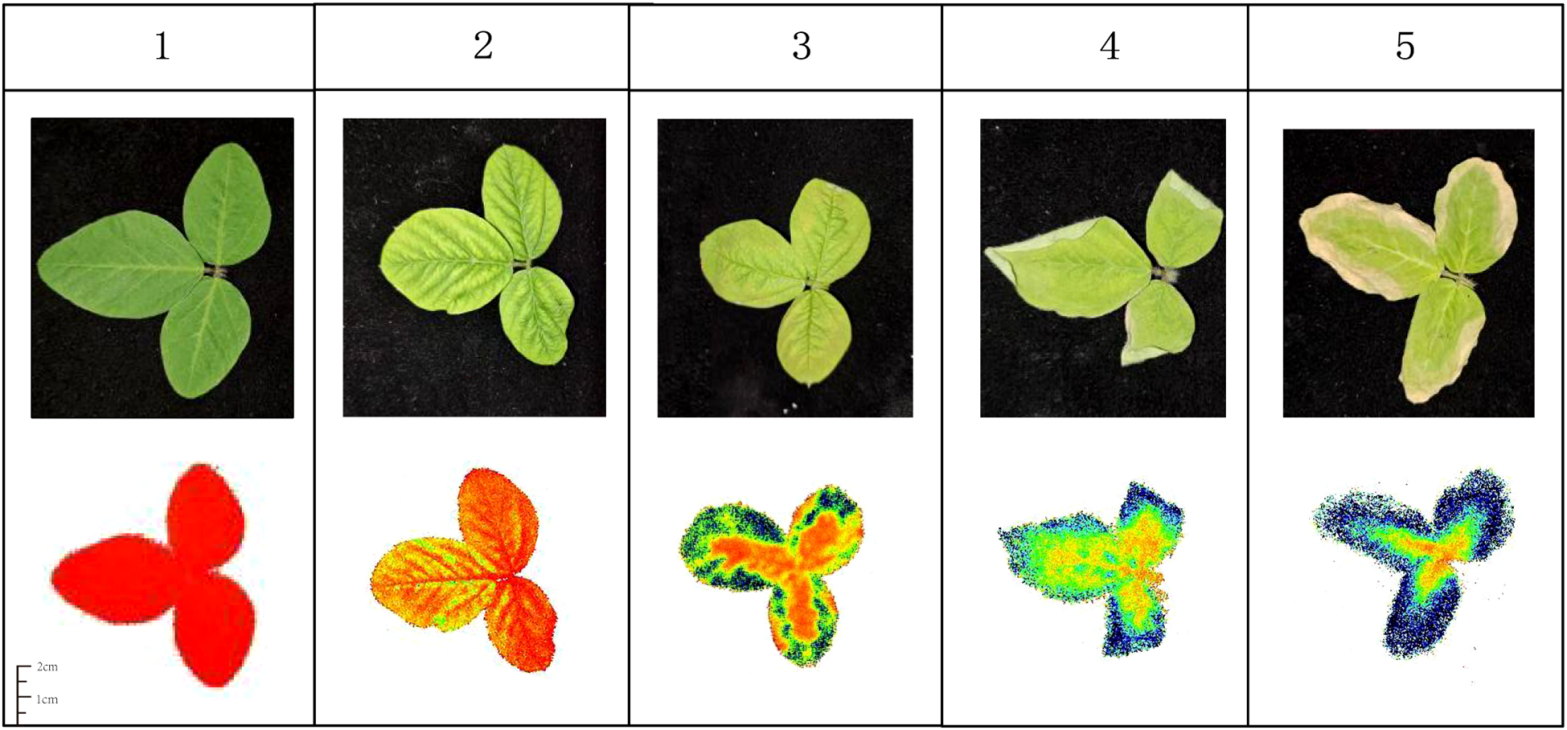
Chlorophyll fluorescence 5 phenotypic data categories. Level 1: Healthy green. Level 2: Slightly lighter, mild tip/edge chlorosis. Level 3: Brown mottling, noticeable chlorosis. Level 4: Brown mottling, severe chlorosis, curled/softened edges. Level 5: Extensive edge yellowing/browning, wilted grayish - white margins.

The image data obtained using the FluorCam instrument has a resolution of 1024 × 768 pixels, with a small leaf area proportion and significant amounts of white space. To enhance data quality, images were cropped to a size of 664 × 664 pixels based on the center of the original images. As shown in **Table 2**, a total of 1231 original images and corresponding text data were collected. The dataset was divided in an 8:1:1 ratio, and data augmentation techniques were applied, including rotations of 90, 180, and 270 degrees as well as horizontal and vertical mirroring. After data augmentation, a total of 7321 images and corresponding text data were generated. The QY_max value serves as a sensitive indicator reflecting the salt stress response of soybean seedlings. The Calinski-Harabasz clustering algorithm was employed to conduct unsupervised optimal cluster partitioning of the relevant text data. As shown in **Figure 3B**, when the data were partitioned into 5 clusters, the within-cluster variance was minimized while the between-cluster variance was maximized, resulting in optimal clustering performance. Combining the phenotypic characteristics related to salt damage levels in soybean seedlings under salt stress, the final classification of soybean salt tolerance was divided into five levels (Jiang et al., 2012; Moon et al., 2023; Zhang et al., 2024). As illustrated in **Figure 4**:Level 1: Healthy green plants with no observed damage, and Fv/Fm values greater than or equal to 0.79.Level 2: Light coloration with no significant defects, slight loss of greenness at leaf tips or edges, and Fv/Fm values between 0.61 and 0.78.Level 3: Leaves exhibit brown spots with symptoms of chlorosis, and Fv/Fm values between 0.46 and 0.60.Level 4: Presence of brown spots, leaves become chlorotic with curled and softened edges, and Fv/Fm values between 0.31 and 0.45.Level 5: Increased areas of yellowing or browning at the leaf edges, with edges wilting to a grayish-white color, and Fv/Fm values between 0.20 and 0.30.

**Table 2:** Sample size of the salt stress dataset.

Label	QY_max	Raw data	Data augmentation
1	≥0.79	255	1465
2	0.61-0.78	159	954
3	0.46-0.6	168	1008
4	0.31-0.45	290	1740
5	0.20-0.30	359	2154

### 2.4 Mm-VitnNet model architecture

**Figure 5:**
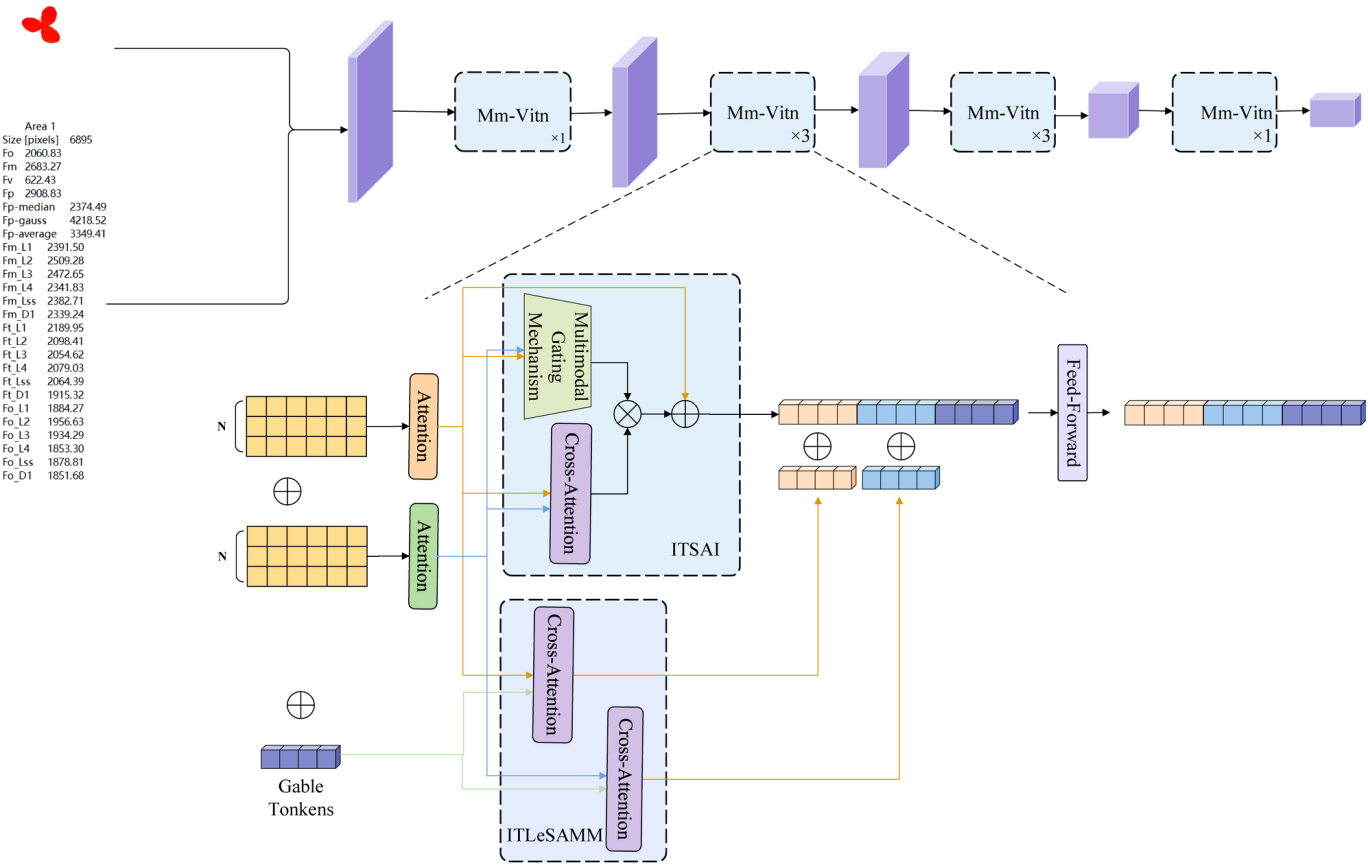
The Mm-VitnNet model architecture processes two types of multimodal data: images and text. The model first extracts features from each modality independently, followed by a cross-modal interaction guided by an image-centric gating mechanism. Additionally, it incorporates learnable tokens to capture long-range feature information for each modality.

The Mm-VitnNet model employs a learnable long-distance cross-modal attention fusion architecture. The input images are uniformly resized to 224 × 224 pixels and subjected to pixel value normalization and standardization preprocessing. Features are extracted and channel expansion is performed through a 3 × 3 convolutional layer, achieving 2× spatial downsampling. This is followed by a max pooling layer that further executes 2× downsampling, ultimately compressing the image feature map size to 56 × 56 pixels. The architecture of the Mm-VitnNet model is illustrated in **Figure 5**, consisting of a backbone structure with four stages. The number of blocks contained in each stage is 1, 3, 3, and 1, respectively. Each block in the first three stages primarily consists of an ITSAI module and an ITLeSAMM module, while the blocks in the fourth stage are composed solely of ITLeSAMM modules.

#### 2.4.1 ITSAI module

**Figure 6:**
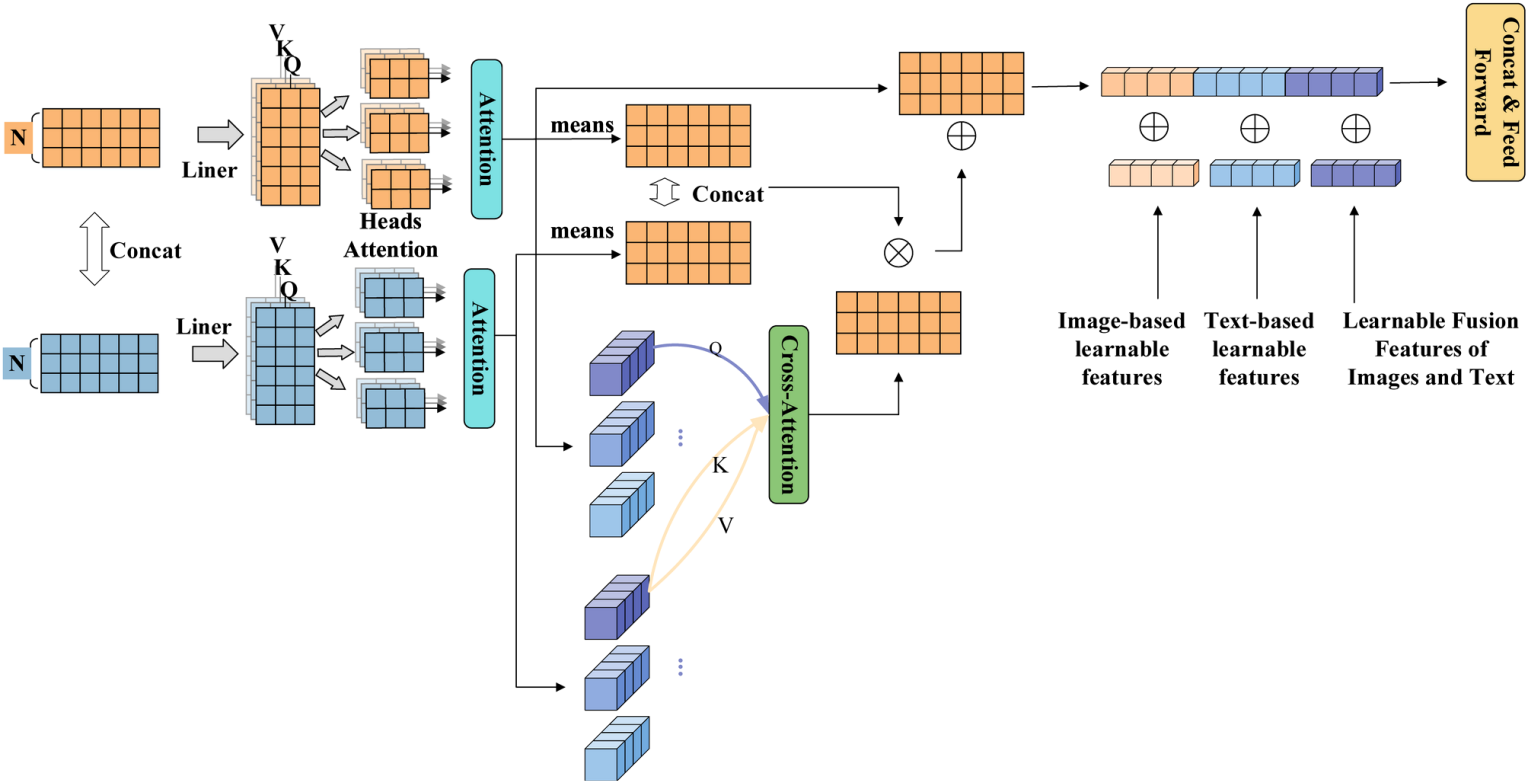
ITSAI module. First, both image and text modalities independently undergo multi-head self-attention mechanisms to learn modality-specific feature information. Subsequently, a cross-modal interaction based on self-attention is performed. Finally, an image-centric gated attention mechanism determines the intensity of the feature fusion between the image and text.

The ITSAI module is the core component of the model that achieves cross-modal semantic alignment between images and text. Its design follows a progressive interaction pattern that includes intra-modal feature enhancement, directed cross-modal retrieval, and adaptive gated fusion. As illustrated in **Figure 6**, the module first uses linear projections to map the input features into query, key, and value vectors. After reshaping and dimensional adjustment, it separates the image features, text features, and learnable global features based on predefined indices. To enhance the internal representations of each modality and reduce cross-modal noise interference, the module performs window-based local self-attention calculations on the image features and global self-attention calculations on the text features, thereby effectively extracting the contextual information from each modality. Subsequently, a guided cross-modal retrieval mechanism is employed, where image features serve as queries and text features act as keys and values. Through attention calculations, the module establishes semantic directional associations from images to text. To further improve the robustness and adaptability of the fusion, the module incorporates a learnable gated weight generation mechanism. Global average pooling is applied to the self-attention-enhanced image and text features. The pooling results are concatenated and fed into two linear layers followed by a ReLU activation, ultimately producing a scalar gating coefficient ranging from 0 to 1. This coefficient is element-wise multiplied with the features obtained from the cross-modal retrieval, allowing for dynamic adjustment of the fusion intensity. Finally, the enhanced features interact with a global memory unit to integrate long-range dependencies. Through this structured design, the module achieves precise, adaptive, and interpretable cross-modal feature fusion while preserving modality specificity.

#### 2.4.2 ITLeSAMM module

**Figure 7:**
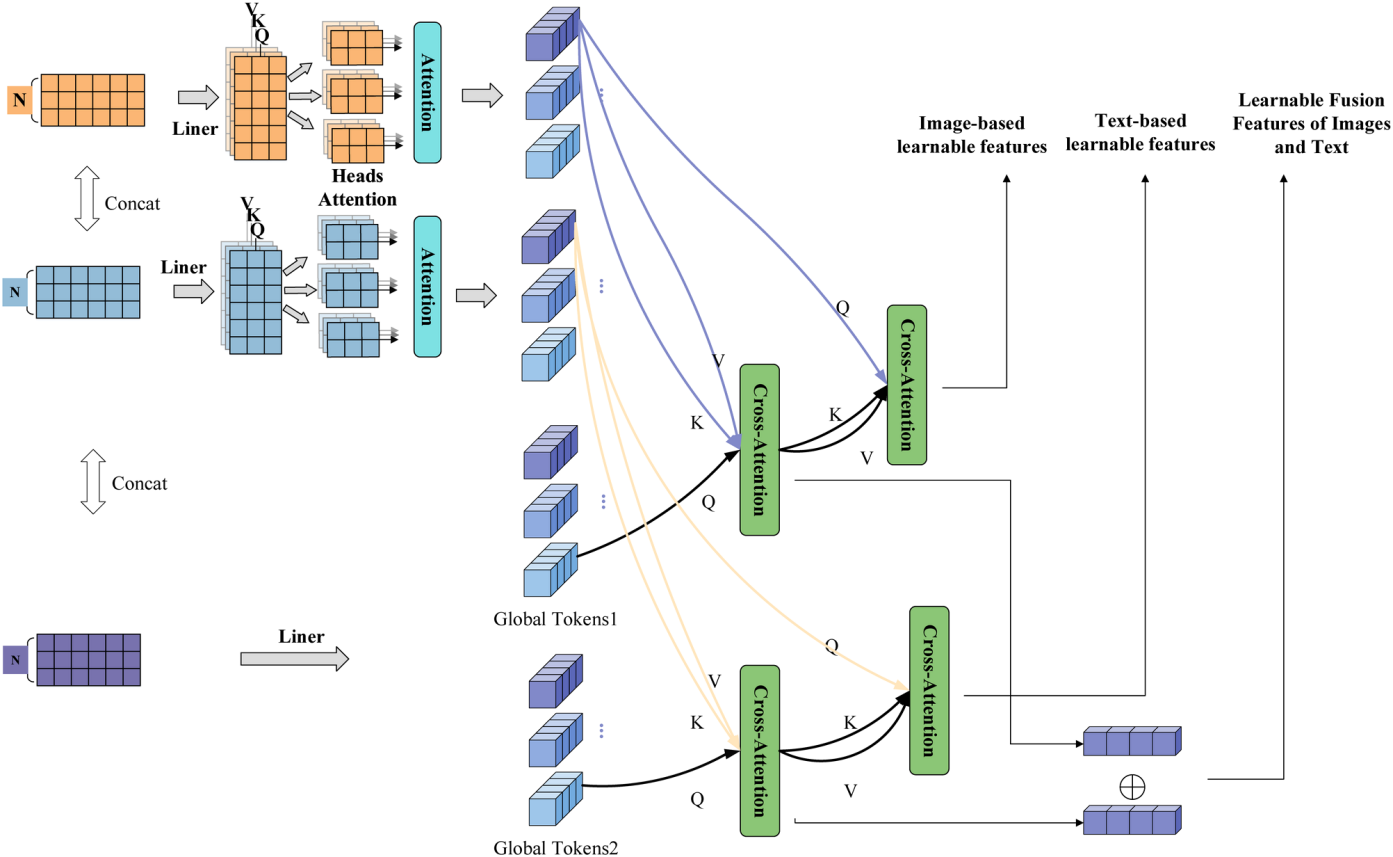
ITLeSAMM modules. This module employs two learnable tokens to capture information from each modality, effectively mitigating inter-modal interference while preserving modality-specific features.

To address the limitations of long-sequence self-attention in modeling local information and the interference caused by remote textual noise in cross-modal fusion, this work introduces a stage-wise global feature aggregation mechanism within the ITLeSAMM module. This mechanism employs two sets of learnable global memory units to model the global semantic information of the image and text modalities, respectively. The core of this mechanism is the introduction of two sets of learnable global memory units (Global Tokens), which serve to extract global feature information for both image and text modalities. During the aggregation phase, each set of tokens interacts with features from the same modality through attention mechanisms: the first set of global tokens acts as queries, with image features serving as keys and values, facilitating self-attention that adaptively focuses on key regions within the image features. This process aggregates to form a feature vector representing the global semantics of the image. Similarly, the second set of global tokens serves as queries, using text features as keys and values, generating the corresponding global text features through self-attention. This design assigns the task of learning long-range dependencies to these dedicated learnable parameters, enabling them to address the challenges associated with long-distance learning and directly learn the global semantic relationships within each modality. The phased strategy of first aggregating within modalities and then utilizing across modalities effectively isolates noise from the initial fusion stage. Image and text features initially complete learning and enhancement within their respective modalities, thereby avoiding the loss of specificity that can occur due to premature cross-modal interaction. Subsequently, the refined and globally informative aggregated features are employed for further cross-modal broadcasting or interaction, which helps ensure the integrity of core information from each modality during the fusion process. Of course, the effectiveness of the global tokens relies on their ability to learn key information from the data. To balance model capacity with training efficiency, experiments set the length of the text global tokens to 256. After concatenating the image and text symbols, dynamic adjustments are made during the four stage levels in line with the changes in the image segment sequence. This approach controls the number of parameters while aiming to equip the tokens with sufficient representational capacity to capture long-range dependencies in the text modality, thereby enhancing the aggregation efficiency and generalization performance of the module overall. **Figure 7** ITLeSAMM modules. This module employs two learnable tokens to capture information from each modality, effectively mitigating inter-modal interference while preserving modality-specific features.

### 2.5 Experimental setup

#### 2.5.1 Experimental platform

All experiments were conducted using the PyTorch 1.10 framework (Python 3.8) and ran on the CUDA 11.8 computational architecture. The hardware environment for the experiments includes an AMD R5-7500F processor, an ASUS RTX 4070TiS graphics card (with 16GB of VRAM), and 32GB of DDR5–6000 memory (ASUS B650-F motherboard).

#### 2.5.2 Training settings

During model training, the batch size was set to 32 and the number of training epochs was set to 200. The Adam optimizer was adopted with an initial learning rate of 0.0001. In addition, a LambdaLR learning rate scheduler was employed, which dynamically adjusted the learning rate according to a cosine annealing strategy, allowing the learning rate to periodically decrease as the number of iterations increased and eventually approach 0.000001. With this parameter configuration, the training process enabled the model to first rapidly explore the parameter space and subsequently fine-tune the optimal solution, making it well suited for the learning task (Zhu et al., 2019; Paymode et al., 2021). The CrossEntropyLoss function was used to compute the training loss, and differentiated class weights were assigned to different categories (with higher weights for classes with fewer samples) to balance their contributions during training and effectively mitigate the impact of class imbalance.

#### 2.5.3 Evaluation metrics

To objectively evaluate the performance of this model, this study intends to use four classification evaluation indicators, namely precision, recall, specificity, and accuracy, for assessment. Their calculation formulas are shown in Equations 1–4.

(1) $$\text{Accuracy}=\frac{\text{TN}+\text{TP}}{\text{TN}+\text{TP}+\text{FN}+\text{FP}}\times 100\%$$

(2) $$\text{Precision=}\frac{\text{TP}}{\text{TN+FP}}\times 100\%$$

(3) $$\text{Recall=}\frac{\text{TP}}{\text{TP+FN}}\times 100\%$$

(4) $$\text{F}1-\text{Measure}=\frac{2\text{TP}}{2\text{TP}+\text{FN}+\text{FP}}\times 100\%$$

In the equation, TP (true positive) denotes correctly classified positive samples, FP (false positive) refers to negative samples misclassified as positive, FN (false negative) indicates positive samples misclassified as negative, and TN (true negative) represents correctly classified negative samples.

## 3 Results

### 3.1 Network models performance analysis

Under identical experimental conditions, this study conducted a multidimensional comparison of the proposed Mm-VitnNet network model with classical convolutional networks [VGG (Simonyan and Zisserman, 2014), ResNet50 (He et al., 2016), and DenseNet (Huang et al., 2017)], lightweight networks (MobileNetV2 (Sandler et al., 2018), ShuffleNetV2 (Ma et al., 2018), EfficientNet (Tan and Le, 2019), and EfficientNetV2 (Tan and Le, 2021)), as well as several Transformer architecture models [ViT (Dosovitskiy et al., 2020) and Swin Transformer (Liu et al., 2021)] and convolutional-Transformer hybrid models [MobileViT (Mehta and Rastegari, 2021)]. The Mm-VitnNet model achieved an accuracy of 98.97%. As shown in **Table 3**, this represents an improvement of 1.16 percentage points over VGG (97.81%), 1.51 percentage points over ResNet50 (97.46%), 1.30 percentage points over DenseNet121 (97.67%), 1.23 percentage points over EfficientNet-b0 (97.74%), and 1.09 percentage points over EfficientNetV2-s (97.88%). Furthermore, the precision, recall, and F1-score of Mm-VitnNet exceeded those of classical CNNs, indicating superior balance and reliability in classifying both positive and negative samples. This suggests that the proposed model demonstrates a more significant capability for identifying samples across different levels of salt tolerance. In comparison with lightweight CNNs and Transformer architectures, Mm-VitnNet outperformed MobileNetV2 (96.64%) by 2.33 percentage points and ShuffleNetV2 (95.21%) by 3.76 percentage points. This highlights that the Mm-VitnNet model can maintain classification performance even under limited computational resources, thereby mitigating some of the accuracy shortcomings associated with lightweight models.

**Table 3:** Comparison of performance across different network models.

Arch.	Model	Accuracy/%	Precious/%	Recall/%	F1-score/%	Parameters/M	FLOPs/G
CNN	VGG	97.81	97.54	97.77	97.65	138.28	15.3
	Resnet50	97.46	96.98	97.33	97.14	23.51	4.13
	MobileNetV2	96.64	96.72	96.58	96.64	2.23	0.326
	ShuffleneV2	95.21	95.26	95.21	95.16	1.26	0.15
	Densenet121	97.67	97.69	97.67	97.67	6.95	2.89
	EfficientNet-b0	97.74	97.77	97.74	97.73	4.01	0.41
	EfficientNetV2-s	97.88	97.88	97.88	97.88	20.18	5.40
Transformer	ViT_b_16	91.72	91.47	91.32	91.38	21.85	4.30
	Swin Transformer_tiny	95.76	95.75	95.76	95.74	28.3	2.97
	MobileViT_S	96.37	96.84	95.99	96.39	4.94	1.46
	(Our)	98.97	98.89	99.05	98.97	10.22	1.84

The ViT model exhibited limitations in capturing global features, resulting in subpar performance for tasks such as identifying soybean salt tolerance levels (accuracy of 91.72%). Although the Swin Transformer showed improvements, the accuracy of the Mm-VitnNet model (98.97%) still surpassed it by 3.21 percentage points. Additionally, the hybrid architecture MobileViT_S (96.37%) lagged behind by 2.60 percentage points. These results demonstrate that the Mm-VitnNet model is well-suited for feature extraction and classification in chlorophyll fluorescence scenarios, exhibiting both stability and efficiency in soybean salt tolerance phenotyping tasks.

The Mm-VitnNet model has a parameter count of 10.22 million, placing it between lightweight CNNs (like MobileNetV2 and ShuffleNetV2) and medium-scale models (like EfficientNet-b0 and MobileViT). Compared to lightweight models (e.g., ShuffleNetV2 with 1.26 million and MobileNetV2 with 2.23 million parameters), it has a larger parameter size yet offers stronger feature expression capability. In comparison to larger models (like ViT_b_16 and Swin Transformer_tiny), it has fewer parameters, making it more lightweight. The model’s computational complexity, measured in FLOPs, is 1.84 billion, which is lower than VGG (15.3 billion), ResNet50 (4.13 billion), and EfficientNetV2-s (5.40 billion), indicating superior computational efficiency. However, it is still higher than lightweight CNNs (e.g., ShuffleNetV2 at 0.15 billion, MobileNetV2 at 0.326 billion, and EfficientNet-b0 at 0.41 billion), highlighting some limitations in computational load. Compared to transformer models, it is lower than ViT_b_16 (4.30 billion) and EfficientNetV2-s (5.40 billion), but higher than Swin Transformer_tiny (2.97 billion) and MobileViT_S (1.46 billion), suggesting that its computational efficiency is at a moderate level among transformer architectures.

**Figure 8:**
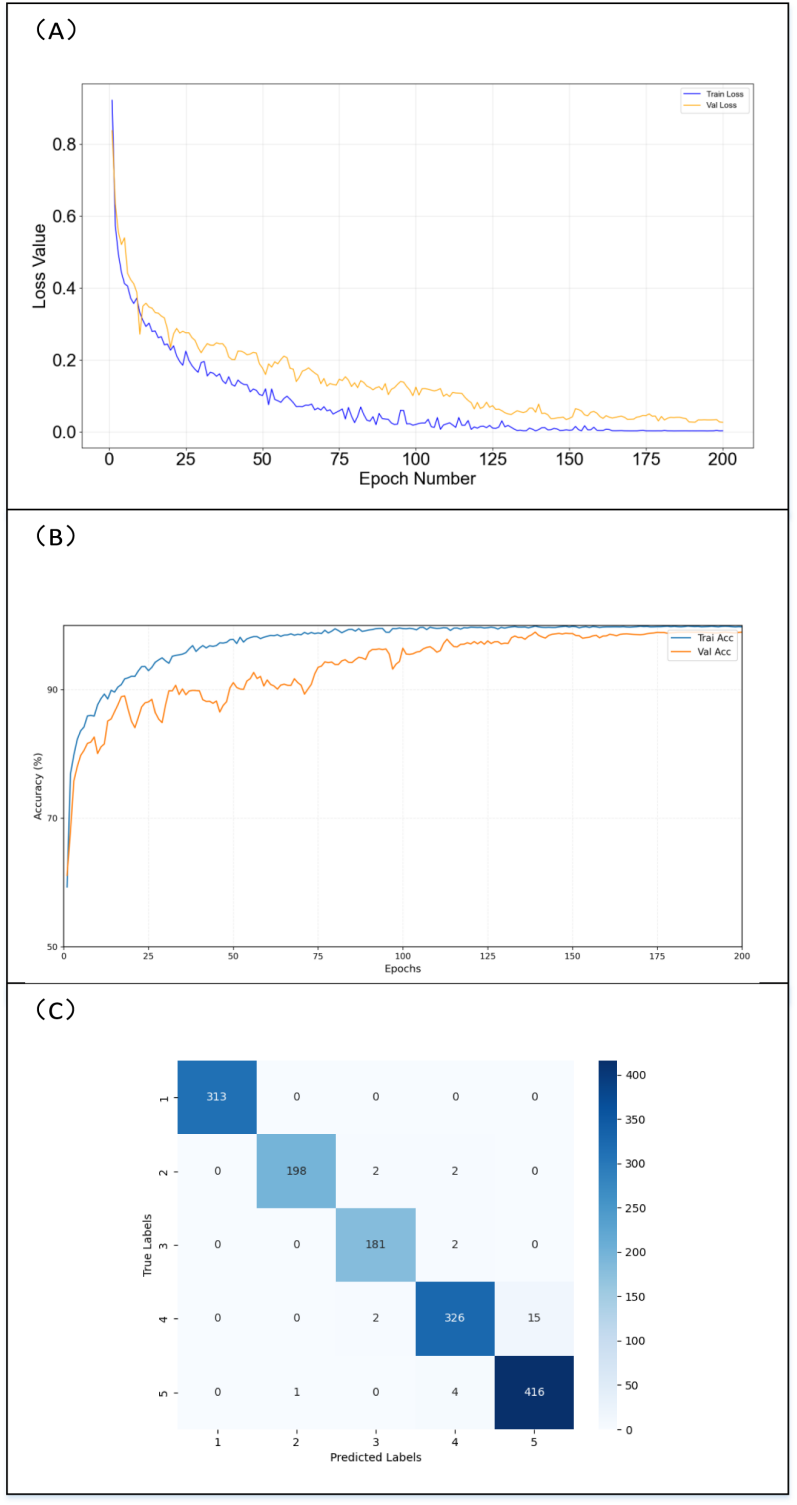
Training and evaluation results of the model. **(A)** Loss visualization. **(B)** Accuracy visualization. **(C)** Confusion matrix visualization.

To comprehensively evaluate the performance of the Mm-VitnNet classification model, a confusion matrix is utilized to illustrate instances where the model misclassifies samples from different categories into other categories. This provides insight into the model’s classification results between the dataset categories. The visualization results of model training and testing are presented in **Figure 8**, where **Figure 8A** shows the loss values over 200 epochs for both training and validation, **Figure 8B** displays the accuracy for training and validation over the same 200 epochs, and **Figure 8C** presents the confusion matrix for model testing. It can be observed from the figures that the model’s recognition bias is primarily at Level 4, with 2 samples misidentified as Level 3 and 15 samples misidentified as Level 5. Overall, the model demonstrates high prediction accuracy across all categories, indicating effective recognition performance.

### 3.2 Ablation analysis

To validate the effectiveness of the model, different combinations of the improved modules were tested to identify the optimal interaction method between images and text. Following this, experimental analyses were conducted on the structural proportions and the selection of the number of principal components from the text PCA. This led to the identification of the overall optimal design of the model and the appropriate number of text inputs used.

#### 3.2.1 Validation of the improved model’s effectiveness

To verify the effectiveness of the Mm-VitnNet module, we first tested the ITSAI and ITLeSAMM modules of the model. The ITSAI module features four structures: ITSAI_b (bidirectional interaction weight selection between images and text), ITSAI_ai (unidirectional interaction between images and text with an image gating mechanism), ITSAI_aip (unidirectional interaction between images and text with both image and text gating mechanisms), and ITSAI_bip (bidirectional interaction weight selection between images and text with both image and text gating mechanisms). These were combined with ITLeSAMM (learnable image and text) to validate the effectiveness of each module. As shown in **Table 4**, the effectiveness of each improved module was verified. The first experimental group constructed a 4-layer ITSAI + ITLeSAMM structure to explore the model architecture. The remaining four experimental groups built 3-layer ITSAI + ITLeSAMM structures with an additional 4th layer of ITLeSAMM. The second group aimed to improve the model architecture while investigating the need to increase the fusion ratio between the interaction-derived feature information and the non-interaction-derived feature information. The third group focused on whether it was possible to extract related unidirectional feature information from images and text based on image data only, adding only image fusion measures. The fourth group added two fusion control mechanisms to explore whether the text control mechanism positively affects model construction, all while maintaining an image-centric approach. The fifth experimental group maintained equal attention to both image and text data while incorporating two gating mechanisms to explore whether text has a similarly positive effect on the task as images do. The exploration of image and text interaction combinations, as well as combinations of image gating and image-text gating, were separately validated. Considering parameters and FLOPs, the combination structure of ITSAI_ai and ITLeSAMM was selected for model construction.

**Table 4:** Performance comparison with attention mechanism.

Model	Accuracy/%	Parameters/M	FLOPs/G
ITSAI_ai + ITLeSAMM*	93.37	10.22	1.84
ITSAI_b + ITLeSAMM	96.85	9.19	1.83
ITSAI_ai + ITLeSAMM	98.36	10.22	1.84
ITSAI_aip + ITLeSAMM	98.70	11.25	1.84
ITSAI_bip + ITLeSAMM	98.91	11.25	1.84

#### 3.2.2 Validation of the effectiveness of the improved structure

After verifying the effectiveness of each module in the model, we explored the relationship between model performance and the structures of the blocks. Initially, different combinations of the number of layers within the stages were tested, evaluating the baseline model, local enhancement model, global enhancement model, and mixed enhancement model. According to the analysis in **Table 5**, the local enhancement model showed a 1.24% increase in accuracy compared to the baseline model, which is significantly higher than the 0.76% improvement observed in the global enhancement model. This indicates that, in this task, the differences between sample categories are primarily reflected in local texture features, making the gains from reinforced local learning more substantial. The mixed enhancement model achieved an accuracy of 98.97%, providing only a 0.13% improvement over the local enhancement model, while the parameter count increased by 27.6% (from 8.01M to 10.22M). This suggests that once local feature learning is sufficiently established, the marginal benefits of global features diminish. Additionally, the parameter scale of the local enhancement model (8.01M) is smaller than that of the global enhancement model (9.66M).To achieve better accuracy, this study selected the mixed enhancement model structure with N = (Zhu et al., 2018; Zhu et al., 2018; Arshad et al., 2025; Arshad et al., 2025) for further analysis.

**Table 5:** Performance comparison based on stage computation proportion.

Model	N_i_	Accuracy/%	Parameters/M	Latency/ms
Baseline Model	(1,1,1,1)	97.60	7.45	1.06
Local Enhancement Model	(1,1,3,1)	98.36	9.66	1.49
Global Enhancement Model	(1,3,1,1)	98.84	8.01	1.41
Hybrid Enhancement Model	(1,3,3,1)	98.97	10.22	1.84

#### 3.2.3 Validation of the effectiveness of the text data quantity

The dataset used in this study consists of both image and text data. Given that the collected text data includes 80 parameters, the excessive number of parameters led to redundancy. To address this issue, we performed dimensionality reduction using Principal Component Analysis (PCA). To fully utilize the text data in the model, we evaluated the effectiveness of using 3, 4, 5, 6, and 7 PCA components as corresponding text data for the image data. Based on the model recognition accuracy presented in **Table 6** and considering practical circumstances, we selected 6 principal components as the usable text data.

**Table 6:** The number of principal components of the model text is selected.

Number of principal components (PCA)	Accuracy/%	Precision/%	Recall/%	F1/%
3	96.85	96.84	97.17	97.00
4	98.91	99.13	99.06	99.09
5	98.29	98.17	98.47	98.31
6	98.97	98.89	99.05	98.97
7	97.81	97.88	97.72	97.85

## 4 Discussion

This study proposes a multimodal recognition network, Mm-VitnNet, for soybean salt tolerance level classification. The model utilizes chlorophyll fluorescence images and text information constructed based on fluorescence parameters for feature extraction and fusion. Experimental results show that Mm-VitnNet achieves superior classification performance compared to single-modality models and traditional fusion methods, validating the effectiveness of the proposed method in mining salt stress-related phenotypic information. Compared to methods based solely on chlorophyll fluorescence features or single-modality deep learning models (Arnob et al., 2025), Mm-VitnNet’s intra-modality self-attention mechanism effectively extracts features from both the image and text modalities. Unlike simple feature concatenation and cross-modal interaction methods (Wang et al., 2026), the cross-modal interaction mechanism of Mm-VitnNet introduces modality-specific tokens and applies local-global broadcast aggregation self-attention mechanisms separately for both image and text. This mechanism effectively mitigates the interference between different modality information during multimodal fusion and the potential dilution of local critical information in long-distance learning, thereby enhancing the stability of feature expression and overall recognition ability while fully learning modality-specific feature information.

Despite the favorable experimental results, this study still has certain limitations. Firstly, the current text modality information is mainly based on chlorophyll fluorescence parameters, lacking a fine description of spatial phenotypic features across different leaf regions, which may limit the exploration of spatial dimension phenotypic information. Secondly, the multimodal data was mainly collected under controlled salt stress conditions, without covering diverse samples from different abiotic stress environments or crops/varieties. Finally, the model’s generalization ability in natural complex environments remains to be further studied and validated. In response to these issues, future research will focus on three aspects: 1) Expanding the dataset to include multiple environments, types of stress, and crop varieties to improve the model’s generalization performance; 2) Introducing fine phenotypic analysis methods based on leaf spatial partitioning to further enrich the multimodal phenotypic information; 3) On the application front, given the more complex phenotypic expression and interference factors in natural environments, future work will involve data collection under conditions that more closely resemble natural complex environments and conduct systematic validation and optimization of the model.

## 5 Conclusion

This study addresses the need for rapid and non-destructive identification of soybean seedling salt tolerance. A multimodal dataset based on chlorophyll fluorescence images and fluorescence parameters was constructed, and a multimodal recognition network, Mm-VitnNet, was proposed. The model effectively integrates visual phenotypic information and physiological parameter data by incorporating intra-modality self-attention and explicit cross-modal interaction mechanisms. Mm-VitnNet outperforms single-modality methods and traditional fusion models in the soybean salt tolerance level classification task. The results indicate that the proposed method provides a stable, non-destructive, and potentially scalable solution for intelligent phenotyping of crop stress tolerance.

## Data Availability

The raw data supporting the conclusions of this article will be made available by the authors, without undue reservation.
